# Supplementary material for: Splice-Junction-Based Mapping of Alternative Isoforms in the Human Proteome
Source: Cell Rep. Author manuscript; Available in PMC 2020 Jan 15. (PMC6961840; doi:10.1016/j.celrep.2019.11.026)
Supplement: 2 [file NIHMS1546469-supplement-2.zip › DF1/PXD010154/Liver/Liver_2_CLTCL1_LQEHFQLQNLGINPANIGFSTLTMESDKFICIR.pdf]

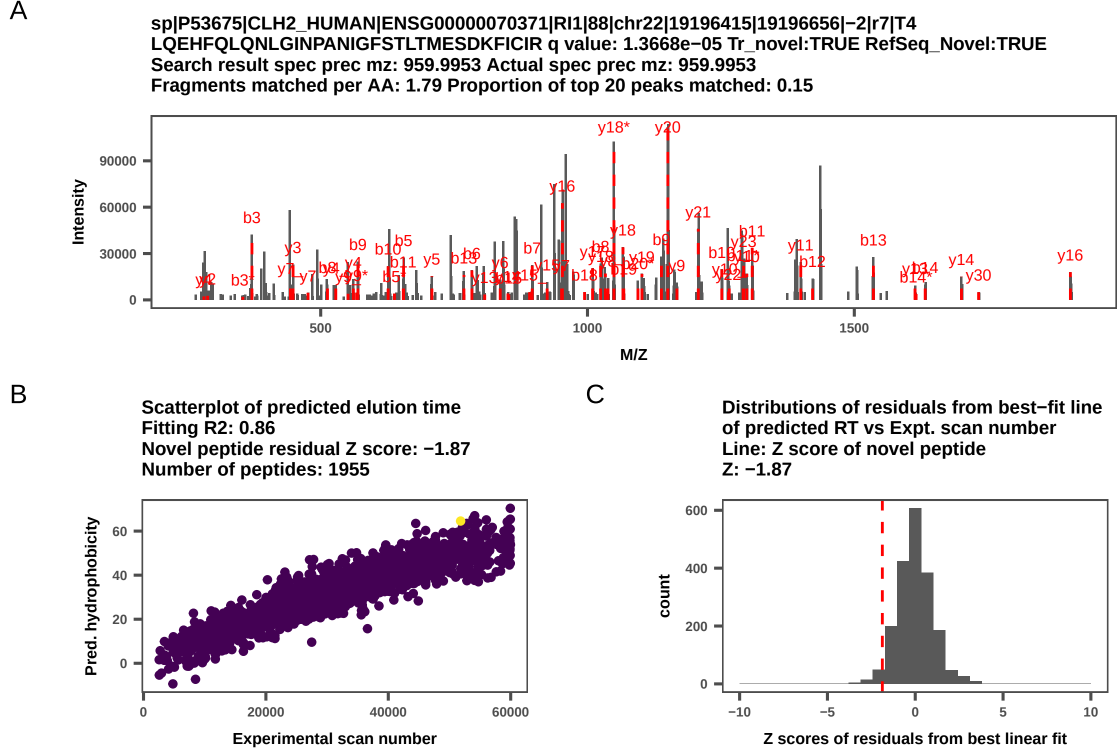
A
sp|P53675|CLH2_HUMAN|ENSG00000070371|RI1|88|chr22|19196415|19196656|−2|r7|T4
LQEHFQLQNLGINPANIGFSTLTMESDKFICIR q value: 1.3668e−05 Tr_novel:TRUE RefSeq_Novel:TRUE
Search result spec prec mz: 959.9953 Actual spec prec mz: 959.9953
Fragments matched per AA: 1.79 Proportion of top 20 peaks matched: 0.15
Intensity
M/Z
B
Scatterplot of predicted elution time
Fitting R2: 0.86
Novel peptide residual Z score: −1.87
Number of peptides: 1955
Pred. hydrophobicity
Experimental scan number
C
Distributions of residuals from best−fit line
of predicted RT vs Expt. scan number
Line: Z score of novel peptide
Z: −1.87
count
Z scores of residuals from best linear fit
